# Disease Stage-Dependent Clinical Impact of *CTLA4* Polymorphism in Multiple Myeloma Treated with Autologous Stem Cell Transplantation

**DOI:** 10.3390/cancers18060963

**Published:** 2026-03-16

**Authors:** Pinar Horum, Katja Seipel, Inna Shaforostova, Martina Bertschinger, Ulrike Bacher, Thomas Pabst

**Affiliations:** 1 Department of Medical Oncology, Inselspital, Bern University Hospital, University of Bern, 3010 Bern, Switzerland; pinar.horum@students.unibe.ch (P.H.); innaivanovna.shaforostova@insel.ch (I.S.); martina.bertschinger@insel.ch (M.B.); 2 Department for Biomedical Research (DBMR), University of Bern, 3008 Bern, Switzerland; veraulrike.bacher@insel.ch; 3 Department of Hematology, Inselspital, Bern University Hospital, University of Bern, 3010 Bern, Switzerland

**Keywords:** multiple myeloma (MM), disease stage (ISS), autologous stem cell transplant (ASCT), progression-free survival (PFS), overall survival (OS), cytotoxic T-lymphocyte-associated protein 4 (*CTLA4*), single-nucleotide polymorphism (SNP)

## Abstract

Background: Clinical outcomes for multiple myeloma are highly variable. Inherited genetic variants of immune regulatory genes can modulate disease susceptibility and clinical outcomes. The germline variant *CTLA4* rs231775 polymorphism may alter T-cell function and affect clinical outcomes. Methods: We conducted a retrospective single-center study including 156 consecutive myeloma patients who underwent first-line ASCT. The patients were stratified according to the *CTLA4* rs231775 genotype and disease stage (ISS I–III). Results: The *CTLA4* rs231775 AA genotype was associated with inferior PFS in ISS I–II and superior PFS in ISS III. In the multivariate analysis, the *CTLA4* rs231775 AA genotype emerged as a potential risk factor in ISS I-II and a potential protective factor in ISS III. Conclusions: This germline *CTLA4* polymorphism may serve as biomarker to refine post-transplant risk stratification and enable personalized treatment management.

## Simple Summary

Despite therapeutic advances, multiple myeloma is incurable, with highly variable clinical outcomes. Inherited variants of immune regulatory genes can modulate disease susceptibility and clinical outcomes. In this study, we investigated a specific polymorphism of the immune checkpoint gene, *CTLA4*, in myeloma patients undergoing autologous stem cell transplantation. The *CTLA4* rs231775 AA genotype was associated with inferior outcomes in early- and intermediate-stage disease and superior outcomes in late-stage disease. The *CTLA4* rs231775 polymorphism may contribute to post-transplant immune regulation, risk stratification, and personalized medicine approaches.

## 1. Introduction

Multiple myeloma (MM) is a plasma cell malignancy originating in the bone marrow with a wide range of clinical symptoms including bone deterioration, hypercalcemia, renal failure, and anemia [1]. Despite recent therapeutic advances, MM remains incurable, and the global disease burden is estimated to rise markedly in the near future [2].

The current MM risk stratification relies on cytogenetic and genomic abnormalities detected by fluorescence in situ hybridization (FISH) and next-generation sequencing (NGS) [3,4]. FISH can detect chromosomal aberrations and remains the standard diagnostic method, as NGS technical approaches are not readily available.

Although MM pathogenesis is driven by somatically acquired gene mutations in plasma cells, the inherited germline variants of immune checkpoint genes can alter disease susceptibility, modulate disease dynamics, and affect clinical outcomes [5,6,7]. The single-nucleotide polymorphism (SNP) rs231775 is a common *CTLA4* germline variant with minor allele frequency (MAF) of 0.36 in the European population. SNPs can be easily detected and are increasingly used in cancer risk stratification and personalized medicine. *CTLA4* rs231775 has been associated with susceptibility to autoimmune disease and cancer [8,9]. Moreover, *CTLA4* rs231775 has been associated with clinical response in DLBCL treated with the CAR T-cell therapy [10]. The rs231775 polymorphism entails the substitution of adenine (A) to guanine (G) at position 49 of the *CTLA4* gene and the associated replacement of the polar amino acid threonine (T17) with the non-polar amino acid alanine (A17), a change that can impair post-translational glycosylation of the *CTLA4* receptor [11] and modify intracellular trafficking, thereby limiting mobilization to the T-cell surface upon activation. The *CTLA4* receptor is a key inhibitory immune checkpoint receptor able to suppress T-cell activation in order to maintain immune homeostasis [12]. In the context of autologous stem cell transplantation (ASCT), T-cells are critical components, and the nature of the *CTLA4* receptor may affect disease control and progression after ASCT [13].

Importantly, the immune regulatory mechanisms are likely modulated by the evolving tumor microenvironment, which becomes progressively more dysregulated [14,15,16]. In early-stage disease (ISS I), the microenvironment is normal with higher proportions of functional immune cells and lower levels of immunosuppressive cells. As disease advances (ISS II), there is a shift in immune cell composition, reflecting early immune evasion. In late-stage disease (ISS III), the microenvironment is characterized by an expansion of immunosuppressive cells and an increase in exhaustion markers on T-cells. The adverse microenvironment in late-stage disease contributes to therapy resistance and inferior survival. In the current study, we investigated the clinical impact of the *CTLA4* rs231775 SNP in MM patients after receiving ASCT, in consideration of disease stage (ISS).

## 2. Patients and Methods

### 2.1. Study Design and Patient Cohort

This retrospective single-center study included MM patients treated at the University Hospital of Bern, Switzerland. All patients signed informed consent, and the study was approved by the local ethics committee of Berne, Switzerland (approval number: 2025-00853; decision date: 24 April 2025). For the purpose of categorizing patients based on their cytogenetics, the following aberrations were included: high-risk abnormalities included t(4;14), t(14;16), t(14;20), del(17p), gain(1q), and *TP53* mutations. Patients with more than one high-risk abnormality were classified as ultra-high-risk, whereas patients with none of the high-risk mutations were considered standard-risk. Where the cytogenetic information was missing, the patients were excluded, using a complete-case approach.

### 2.2. CTLA4 Gene Analysis

*CTLA4* gene sequences were analyzed as previously described [10]. The *CTLA4* geno-type frequencies were analyzed on Hardy–Weinberg equilibrium calculator [17] to compare the observed genotype frequencies in the patient cohort to the allele frequencies in the general European population, as reported previously [18].

### 2.3. Assessment and Endpoints

The response to treatments was assessed using the International Myeloma Working Group (IMWG) response criteria [19]. The primary study endpoints were PFS and OS. PFS was defined as the time between ASCT and either first relapse or death. OS was defined as the time between ASCT and death, irrespective of the cause. The patients lost to follow-up were considered event-free at the time of the analysis. The data were collected until July 2025.

### 2.4. Statistical Analysis

Homogeneity between the genotype groups in categorical and non-parametric variables were evaluated using the Chi-square test or the Mann–Whitney U test. The log-rank test was utilized to compare the Kaplan–Meier curves for PFS and OS. GraphPad Prism, version 10.5.0, was used to perform statistical analyses. A two-sided *p*-value of 0.05 or lower was considered as statistically significant. The multivariate analysis was conducted using the Cox proportional hazards model in R, version 4.4.2. (2024). The median follow-up time was calculated using the method described by Schemper and Smith [20]. The *CTLA4* rs231775 genotype was coded as a binary variable, comparing homozygous major allele carriers (AA) versus carriers of at least one minor allele (GG + AG). This dominant genetic model was chosen to improve statistical stability, given the limited observed frequencies of GG homozygotes. Although an additive three-genotype model was conducted, the dominant model was retained for subsequent multivariate analyses.

## 3. Results

### 3.1. Clinical Characteristics at Baseline

The study included 156 myeloma patients who received induction therapy, followed by high-dose chemotherapy (HDCT) and autologous stem cell transplant (ASCT) in the years 2003–2024 at the University Hospital Bern. The *CTLA4* gene variant rs231775 (NC_000002.12:g.203867991A>G; NP_001032720.1:p.Thr17Ala) was prevalent at MAF 0.36 in the myeloma cohort, same as in the European population (χ^2^ = 0.1, *p* = 0.75). A total of 65 patients (42%) carried two major alleles (AA) encoding *CTLA4* T17, 70 patients (42%) had one minor allele (AG) encoding *CTLA4* T17A, 21 patients (13%) carried two minor alleles (GG) encoding *CTLA4* A17. In the following analysis, two genetic subgroups were compared: patients carrying two major alleles of the *CTLA4* rs231775 polymorphism (AA) versus all others, AA vs. GG + AG. The baseline characteristics were evaluated for the whole patient cohort and for the two *CTLA4* genetic subgroups (Table 1). The median age at diagnosis was 58 years, and the male/female ratio was 2, with equal distribution in both subgroups. The median year of diagnosis was 2017 (range 2003–2024). ISS classifies the disease stage according to serum β2M and serum albumin levels. ISS staging, paraprotein subtypes, and cytogenetics were determined at the initial diagnosis, with differential distribution in the two genetic subgroups: ISS I and standard cytogenetic risk were more prevalent in the GG + AG group, and ISS II and high-risk cytogenetics were more prevalent in the AA group (*p* = 0.013 and 0.044), with equal prevalence of ISS III and ultra-high-risk cytogenetics in both subgroups. Paraprotein subtypes were equally distributed with prevalence of IgG heavy chain (60%) and IgK light chain (67%). Hypercalcemia, renal insufficiency, osteolytic lesions, and the extent of bone marrow infiltration were similarly prevalent in the two subgroups. Anemia was more prevalent in the AA group (*p* = 0.044). *CTLA4* allele frequencies were recalculated according to the ISS staging.

### 3.2. Disease Features and Treatments

All 156 patients received induction therapy, followed by high-dose chemotherapy (HDCT) and autologous stem cell transplant (ASCT). The treatment characteristics were analyzed in the entire cohort and according to *CTLA4* genotype AA vs. GG + AG (Table 2). Induction therapy consisted of bortezomib and dexamethasone (Vd), sometimes in combination with lenalidomide (VRd) and daratumumab (Dara-VRd), or in combination with cyclophosphamide (VCd). Before HDCT/ASCT, 12% of the patients were in complete remission (CR), 38% were in good partial response (VGPR), and 32% were in partial response (PR). High-dose chemotherapy (HDCT) consisted of melphalan, sometimes in combination with bendamustine (BenMel) or treosulfan (Treo-Mel). The treatment characteristics were balanced in the *CTLA4* genetic subgroups, with differences in the HDCT settings. In the AA group, almost half of the patients received Ben-Mel or Treo-Mel, whereas most of the GG + AG group received melphalan alone (*p* = 0.020). Most of the patients underwent a second HDCT/ASCT and 36% received additional radiation therapy, with equal distributions in the *CTLA4* genetic subgroups.

### 3.3. Treatment Outcomes—Univariate Analysis

**Figure 1:**
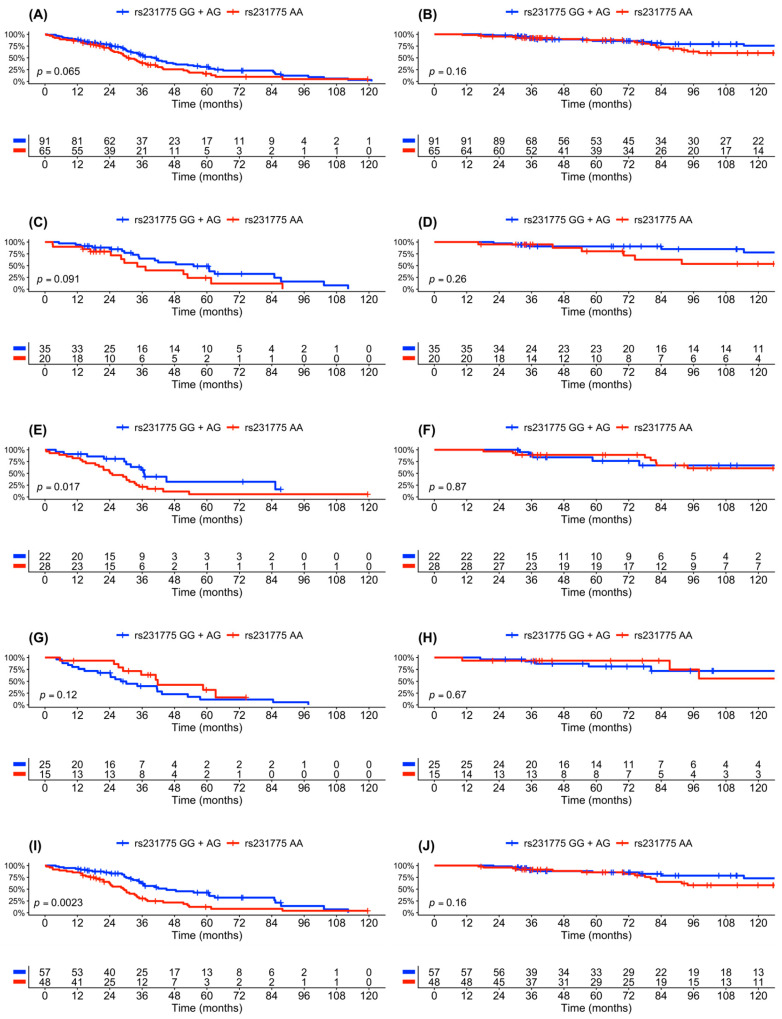
Clinical outcomes in myeloma patients according to *CTLA4* rs231775 genotype and disease stage. Progression-free survival (**A**,**C**,**E**,**G**,**I**) and overall survival (**B**,**D**,**F**,**H**,**J**) according to *CTLA4* rs231775 genotype (GG + AG vs. AA) in the entire patient cohort (**A**,**B**), in patients with early stage disease ISS I (**C**,**D**), intermediate stage disease ISS II (**E**,**F**), late stage disease ISS III (**G**,**H**), and combination of early and intermediate stage disease ISS I–II (**I**,**J**).

The treatment outcomes were analyzed according to *CTLA4* genotype and ISS stage (Figure 1). In the entire cohort, inferior survival outcomes were associated with patients carrying two major alleles of *CTLA4* rs231775 (AA), with median PFS of 30 vs. 40 months (*p* = 0.065) and 8-year OS rate of 60% vs. 80% (*p* = 0.16) (Figure 1A,B). To address the impact of disease stage on treatment outcomes, the subsequent analysis was stratified to ISS I (Figure 1C,D), ISS II (Figure 1E,F), and ISS III (Figure 1G,H). AA carriers in early-intermediate stage disease (ISS I-II) had inferior survival outcomes (Figure 1I,J), whereas no significant association was observed in late-stage disease (ISS III) (Figure 1G,H). A more quantitative representation of the treatment outcomes is presented in Table 3. CR, relapse, and death rates were similar in the two genetic subgroups. In patients with early-intermediate stage disease (ISS I-II), the AA genotype was associated with a shorter median PFS compared with the GG + AG group (29 vs. 45 months; HR 2.05, 95% CI: 1.28–3.30; *p* = 0.003). A similar trend was observed for ISS I disease alone (34 vs. 56 months; HR 1.43, 95% CI: 0.98–2.08; *p* = 0.065). In late stage disease (ISS III), patients carrying the AA genotype showed longer PFS compared with the GG + AG genotype group (40 vs. 30 months; HR 0.54, 95% CI: 0.24–1.20; *p* = 0.12). To provide transparency regarding subgroup sizes and event distribution underlying the ISS stratified analyses, patient numbers and progression events were summarized for each ISS stage and *CTLA4* rs231775 genotype subgroup (Table 4).

**Figure 2:**
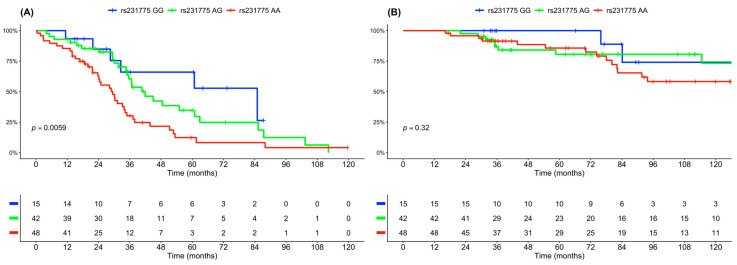
Superior outcomes in myeloma patients with *CTLA4* rs231775 genotype GG in ISS I-II. Progression-free survival (**A**) and overall survival (**B**) in ISS I–II patients according to *CTLA4* rs231775 genotype (GG vs. AG vs. AA).

To address differential impact of heterozygote (AG) and homozygote carriers, an additional analysis in all three genetic subtypes was included. Here, clinical outcomes were superior in the subgroup of patients carrying two minor alleles of *CTLA4* rs231775 (GG) in early-intermediate stage disease (median PFS of 84 vs. 40 vs. 30 months, *p* = 0.0059) (Figure 2). A dose-dependent effect of *CTLA4* rs231775 was indicated, with PFS of the heterozygous group (AG) falling between the two homozygous groups (AA and GG).

### 3.4. Treatment Outcomes—Multivariate Analysis

Due to missing cytogenetic data, the multivariate Cox proportional hazards model including cytogenetic risk was performed in 105 patients. In the multivariate analysis adjusting for sex, age at diagnosis, high-risk cytogenetics, and ISS stage, the *CTLA4* rs231775 polymorphism was significantly associated with PFS after ASCT, with evidence of effect modification by the ISS stage (Table 5). The reference groups consisted of the carriers of the GG + AG genotype, patients with female sex, those with standard-risk cytogenetics, and those with ISS stage I–II disease. High-risk cytogenetics comprised high-risk and ultra-high-risk aberrations. Within the reference ISS group, patients carrying the rs231775 AA genotype had a significantly increased risk of progression compared with GG + AG carriers (HR 2.1, 95% CI: 1.2–3.8; *p* = 0.012). Conversely, within the GG + AG reference group, ISS stage III was significantly associated with inferior PFS (HR 2.1, 95% CI: 1.1–4.1; *p* = 0.028). To address the *CTLA4* variant-dependent effects on PFS observed in the univariate analysis of ISS I-II vs. ISS III, the interaction between rs231775 and ISS stage was analyzed in a separate model. A significant interaction emerged for the AA genotype and ISS III for PFS (HR = 0.2, *p* = 0.01), indicating that the prognostic impact of the AA genotype differed according to disease stage. Sex, age at diagnosis, and high-risk cytogenetics were not significantly associated with PFS.

*CTLA4* rs231775 polymorphism and ISS stage were not significantly associated with OS after ASCT. Within the reference ISS group, male patients had a significant lower death risk compared to female patients (HR 0.3, 95% CI: 0.1–0.7; *p* = 0.008), and patients with high-risk cytogenetics had a higher death risk than standard-risk patients (HR 3.6, 95% CI: 1.6–8.3; *p* = 0.003).

The concordance index of the PFS multivariate model was 0.61, and standard error was 0.035, with likelihood ratio being *p* = 0.05, indicating a model with relatively weak discriminative ability, commonly seen in clinical prognosis studies. The concordance index of the OS multivariate model was 0.68, and standard error was 0.06, with likelihood ratio being *p* = 0.01, indicating a model with moderate discrimination performance in predicting clinical outcome. The proportional hazards assumption was tested using Schoenfeld residuals and was not violated for any of the analyzed covariates and for the overall model for both PFS and OS (*p* > 0.05), indicating that the hazard rate of an individual is relatively constant over time.

Given the significant differences in conditioning regimes between the different genotype groups, a sensitivity analysis was performed in the subgroup of 65 patients who received high-dose melphalan before ASCT and for whom complete data were available (Table 6). In this subgroup, the *CTLA4* rs231775 AA genotype remained significantly associated with inferior PFS (HR 2.1, 95% CI: 1.1–4.2; *p* = 0.029) but was not significantly associated with OS. Male sex was associated with improved OS (HR 0.2, 95% CI: 0.09–0.6; *p* = 0.004), whereas no significant association was observed for PFS. High-risk cytogenetics were significantly associated with both inferior OS (HR 2.9, 95% CI: 1.1–7.5; *p* = 0.026) and PFS (HR 1.9, 95% CI: 1.0–3.6; *p* = 0.042). ISS stage III was not significantly associated with OS but trended towards significance for inferior PFS (HR 2.0, 95% CI: 0.9–4.3; *p* = 0.068). The interaction term between the rs231775 AA genotype and ISS III stage remained significant for PFS (*p* = 0.046). The PFS model demonstrated a concordance index of 0.611 (SE = 0.043) and statistical significance in the likelihood ratio test (*p* = 0.05). The OS model demonstrated a concordance index of 0.682 (SE = 0.062) and statistical significance in the likelihood ratio test (*p* = 0.04). Again, the proportional hazards assumption was assessed using Schoenfeld residuals and was not violated for any of the analyzed covariates and for the overall model for both PFS and OS (*p* > 0.05).

## 4. Discussion

In the studied cohort of multiple myeloma patients, the *CTLA4* rs231775 polymorphism was present at allele frequencies comparable to those observed in the general European population, indicating no association of the *CTLA4* genotype with disease susceptibility. However, there was a disease stage-dependent association of treatment outcomes after ASCT. In early-intermediate stage disease (ISS I-II), the rs231775 AA genotype was significantly associated with inferior PFS, indicating that the rs231775 polymorphism may represent a prognostic marker in this clinical setting. In contrast, in late-stage disease (ISS III), the rs231775 AA associated with superior PFS. Although interaction terms in Cox models have been valuable for identifying context-specific effects of certain markers [21,22,23], they may exaggerate deviations from additive effects on log scale, particularly when subgroup sizes are small. Therefore, the absence of inferior effects of the AA genotype in ISS III patients may either reflect limited statistical power or indicate that the impact of the *CTLA4* genetic variant is less pronounced in late-stage disease. An earlier publication investigating *CTLA4* variants in MM also reported that their prognostic relevance becomes more evident when stratified by the ISS stage [6], supporting the possibility that disease stage modifies the clinical impact of rs231775 polymorphism.

The observed effects are plausible in the context of ASCT, which induces profound and dynamic changes in the immune environment beyond cyto-reduction. Immediately after transplant, there is marked lympho-depletion, followed by homeostatic proliferation and immune reconstitution. This process is characterized by disruption of the bone marrow microenvironment, which transiently enhances antitumor immunity but also creates vulnerability to immune escape [24,25]. In this setting, T-cells are critical for anti-myeloma immunity, but their effectiveness is limited by exhausted phenotypes, which are associated with relapse [26]. Germline variation in *CTLA4* may therefore play a role in the post-transplant period. In early-intermediate stage disease (ISS I-II), altered *CTLA4* in rs231775 AA carriers may contribute to insufficient antitumor response and earlier relapse. In ISS III, the interplay between high tumor burden and immune exhaustion may obscure genotype-specific effects, consistent with the lack of significant association in this subgroup.

At the molecular level, rs231775 AA genotype encodes threonine in the *CTLA4* signal-peptide, while the GG genotype encodes alanine. This change can alter the intracellular dynamics of the receptor, including post-translational routing, intracellular storage, the mobilization to the cell surface, and the timing of subsequent degradation [8,11]. Such alterations can affect T-cell inhibitory signaling and immune responsiveness. Consistent with this concept, the associations between the rs231775 AA genotype and inferior survival outcomes have also been reported in other immune-dependent treatment settings, such as diffuse large B-cell lymphoma treated with CAR T-cell therapy [10].

The role of *CTLA4*-mediated immune regulation may also have implications for immunotherapeutic strategies in MM. Therapies like immune checkpoint inhibitors (ICIs) have not demonstrated significant activity as a single agent in MM treatment [27], as the microenvironment may be characterized by dysfunctional T-cells with features of immuno-senescence and exhaustion, which are not easily reversed by checkpoint blockade [28]. Nevertheless, ICIs are still promising therapies, as they can reinvigorate the immune system in specific subgroups of post-transplant patients [29]. Our findings raise the possibility that patients with early-intermediate disease stage, in whom immune competence is less severely impaired, may represent a subgroup in which a decreased *CTLA4* expression could be more effective. However, such considerations remain exploratory and require validation.

The study cohort comprised transplant-eligible patients diagnosed across different diagnostic and therapeutic eras, with a median year of diagnosis of 2017. Diagnostic criteria for MM were expanded in 2014 to include more disease-defining bio-markers and cytogenetic stratification using FISH became more standardized over time, with high-risk features guiding therapy selection [30]. As a result of these changes, uniformly documented and standardized cytogenetic data were unavailable for half of the study cohort, which represents an inherent limitation of this retrospective analysis. Where cytogenetic risk was included as a variable, a complete-case approach in the multivariate analyses was conducted. Although this reduced the effective sample size, the models still showed acceptable predictive performance and met the proportional hazards assumptions.

Similar transformations are seen in therapeutic strategies. Traditionally, induction therapy consisted of triplet regimens followed by high-dose melphalan conditioning and lenalidomide maintenance, while more recent approaches incorporate anti-CD38 monoclonal antibodies into induction and, in selected cases, into maintenance therapy [31]. Our patient cohort reflects these trends in the received treatments. Markedly, the HDCT regimens varied significantly with melphalan alone versus melphalan combinations and represent a potential confounder. As melphalan is the standard of care prior to ASCT, we performed a sensitivity analysis restricted to patients conditioned with melphalan-only. Within this more homogeneous subgroup, the *CTLA4* rs231775 AA genotype remained significantly associated with inferior PFS in early-intermediate disease, indicating that the impact was not solely driven by differences in conditioning regimens.

The study was limited by the sample size, retrospective single-center design, and long study period, which may have introduced a selection and time bias. The absence of a statistically significant effect on OS may be attributable to prolonged follow-up duration and different post-relapse therapies, potentially diluting genotype-related differences. Interestingly, in multivariate models for OS, the male sex was associated with significantly improved survival. This finding appears counterintuitive, as a previous study showed identical prognosis for female and male patients after ASCT [32]. In this context, our observation of superior OS in male patients may reflect genotype-specific characteristics but should be interpreted cautiously and as hypothesis-generating rather than definitive.

Overall, our findings suggest that the *CTLA4* rs231775 polymorphism may represent a clinically relevant prognostic marker in MM undergoing ASCT, particularly in early-intermediate stage disease (ISS I-II). Furthermore, while the absence of a consistent effect in late-stage disease (ISS III) may be due to limited statistical power, it may also indicate that the profound immune dysregulation of advanced disease diminished the influence of germline variants. Prospective validation in larger, molecularly well-described cohorts will therefore be needed to confirm these findings and determine whether *CTLA4* rs231775 genotyping could contribute to improved risk stratification.

## 5. Conclusions

The germline variant *CTLA4* rs231775 AA genotype is associated with inferior PFS in patients with MM after ASCT, particularly in early-intermediate stage disease (ISS I-II). These results highlight the importance of *CTLA4*-related immune regulation in a post-transplant setting and support further investigation of rs231775 as a biomarker to refine risk stratification and personalize post-ASCT management.

## Figures and Tables

**Table 1:** Baseline clinical characteristics.

		rs231775		
Parameter	Total	GG + AG	AA	*p*-Value
Patients, *n* (%)	156 (100)	91 (100)	65 (100)	
Median age at diagnosis, years (range)	58 (33–77)	58 (39–72)	58 (33–77)	0.87
Male sex, *n* (%)	105 (67)	62 (68)	43 (66)	0.79
Sex ratio (m/f)	2.06	2.14	1.95	
Disease stage, *n* (%)				0.039
ISS I	55 (35)	35 (38)	20 (31)	0.32
ISS II	50 (32)	22 (24)	28 (43)	0.013
ISS III	40 (26)	25 (27)	15 (23)	0.53
Unknown	11 (7)	9 (10)	2 (3)	-
Paraprotein subtype, *n* (%)				0.68
IgG	93 (60)	56 (62)	37 (57)	0.56
IgA	31 (20)	18 (20)	13 (20)	0.97
IgM	1 (1)	1 (1)	0 (0)	-
Light chain-only	31 (20)	16 (18)	15 (23)	0.39
Lambda light chain	52 (33)	30 (33)	22 (34)	0.91
Kappa light chain	104 (67)	61 (67)	43 (66)	0.91
FISH				0.17
Ultra-high-risk ^a^	14 (9)	8 (9)	6 (9)	0.92
High-risk ^b^	42 (27)	19 (21)	23 (35)	0.044
Standard-risk	49 (31)	34 (37)	15 (23)	0.058
Unknown	51 (33)	30 (33)	21 (32)	-
Hypercalcemia ^c^, *n* (%)	19 (12)	12 (13)	7 (11)	0.65
Renal insufficiency ^d^, *n* (%)	19 (12)	13 (14)	6 (9)	0.34
Anemia ^e^, *n* (%)	42 (27)	19 (21)	23 (35)	0.044
Osteolytic lesions, *n* (%)	111 (71)	66 (73)	45 (69)	0.65
Bone marrow infiltration, % (range)	70 (10–100)	70 (10–100)	65 (10–100)	0.38

*n* (%): percentages are column-based and refer to the respective group totals; ISS: International Staging System; ^a^ ≥2 of t(4;14), t(14;16), t(14;20), del(17p), gain(1q), or *TP53* mutation; ^b^ 1 of t(4;14), t(14;16), t(14;20), del(17p), gain(1q), or *TP53* mutation; ^c^ calcium > 2.6 mmol/L; ^d^ serum creatinine > 177 µmol/L; ^e^ hemoglobin < 110 g/L.

**Table 2:** Treatment characteristics.

		rs231775		
Parameter	Total	GG + AG	AA	*p*-Value
Patients, *n* (%)	156 (100)	91 (100)	65 (100)	
Induction therapy, *n* (%)				
VRd	69 (44)	42 (46)	27 (42)	0.57
Dara-VRd	14 (9)	9 (10)	5 (8)	0.64
VCd	44 (28)	22 (24)	22 (34)	0.19
VTd	5 (3)	2 (2)	3 (5)	0.39
VAd	5 (3)	3 (3)	2 (3)	0.94
Vd	19 (12)	13 (14)	6 (9)	0.34
Remission status before ASCT, *n* (%)				
PD	3 (2)	2 (2)	1 (2)	0.77
SD	25 (16)	15 (16)	10 (15)	0.85
PR	50 (32)	29 (32)	21 (32)	0.95
VGPR	59 (38)	35 (38)	24 (37)	0.84
CR	19 (12)	10 (11)	9 (14)	0.59
HDCT, *n* (%)				0.04
melphalan	96 (62)	63 (69)	33 (51)	0.02
BenMel	23 (15)	9 (10)	14 (22)	0.04
TreoMel	37 (24)	19 (21)	18 (28)	0.32
Median number of ASCT, *n* (range)	2 (1–4)	2 (1–4)	2 (1–4)	0.60
Radiation therapy, *n* (%)	56 (36)	33 (36)	23 (35)	0.91

*n* (%): percentages are column-based and refer to the respective group totals; VRd: bortezomib, lenalidomide, dexamethasone; Dara-VRd: daratumumab, bortezomib, lenalidomide, dexamethasone; VCd: bortezomib, cyclophosphamide, dexamethasone; VTd: bortezomib, thalidomide, dexamethasone; VAd: bortezomib, doxorubicin, dexamethasone; Vd: bortezomib, dexamethasone; ASCT: autologous stem cell transplantation; PD: progressive disease; SD: stable disease; PR: partial remission; VGPR: very good partial remission; CR: complete remission; HDCT: high-dose chemotherapy for first ASCT; BenMel: bendamustine, melphalan; TreoMel: treosulfan, melphalan.

**Table 3:** Clinical outcomes, univariate analysis.

		rs231775			
Parameter	Total	GG + AG	AA	HR (95% CI)	*p*-Value
Patients, *n* (%)	156 (100)	91 (100)	65 (100)		
CR, *n* (%)	91 (58)	51 (56)	40 (62)		0.49
Relapse, *n* (%)	112 (72)	63 (69)	49 (75)		0.34
Death, *n* (%)	41 (26)	20 (22)	21 (32)		0.15
PFS, ISS I-III, median	34	40	30	1.41 (0.95–2.09)	0.091
PFS, ISS I, median	43	56	34	1.43 (0.98–2.08)	0.065
PFS, ISS II, median	29	36	24	2.28 (1.14–4.56)	0.017
PFS, ISS I-II, median	36	45	29	2.05 (1.28–3.30)	0.003
PFS, ISS III, median	36	30	40	0.54 (0.24–1.20)	0.12

*n* (%): percentages are column-based and refer to the respective group totals; CR: complete remission; PFS: progression-free survival; ISS: International Staging System; *n*: number.

**Table 4:** Patient numbers and event counts in each ISS × genotype subgroup.

ISS	rs231775	*n*	Events, *n* (%)	5-Year PFS Rate (%)
I	GG + AG	35	21 (60)	49
I	AA	20	13 (65)	24
II	GG + AG	22	12 (55)	32
II	AA	28	25 (89)	6
III	GG + AG	25	21 (84)	11
III	AA	15	9 (60)	32

ISS: International Staging System; *n*: number; PFS: progression-free survival.

**Table 5:** Clinical outcomes-multivariate analysis.

Predictors	OS		PFS	
	HR (95% CI)	*p*-Value	HR (95% CI)	*p*-Value
rs231775 AA	1.3 (0.5–3.1)	0.577	2.1 (1.2–3.8)	0.012
Sex, male	0.3 (0.1–0.7)	0.008	1.4 (0.7–2.5)	0.307
Age at diagnosis	1.0 (1.0–1.0)	0.585	1.0 (1.0–1.0)	0.985
High-risk cytogenetics	3.6 (1.6–8.3)	0.003	1.6 (0.9–1.0)	0.083
ISS III	1.2 (0.4–3.8)	0.706	2.1 (1.1–4.1)	0.028

OS: overall survival; PFS: progression-free survival; HR: hazard ratio; CI: confidence interval; ISS: International Staging System.

**Table 6:** Clinical outcomes in melphalan-only-multivariate analysis.

Predictors	OS		PFS	
	HR (95% CI)	*p*-Value	HR (95% CI)	*p*-Value
rs231775 AA	1.2 (0.4–3.3)	0.705	2.1 (1.1–4.2)	0.029
Sex, male	0.2 (0.09–0.6)	0.004	0.7 (0.3–1.4)	0.296
Age at diagnosis	1.0 (1.0–1.0)	0.333	1.0 (1.0–1.0)	0.384
High-risk cytogenetics	2.9 (1.1–7.5)	0.026	1.9 (1.0–3.6)	0.042
ISS III	1.5 (0.5–4.9)	0.466	2.0 (0.9–4.3)	0.068

OS: overall survival; PFS: progression-free survival; HR: hazard ratio; CI: confidence interval; ISS: International Staging System.

## Data Availability

The data presented in this study are available on request from the corresponding author.
